# MutS and MutL Are Dispensable for Maintenance of the Genomic Mutation Rate in the Halophilic Archaeon *Halobacterium salinarum* NRC-1

**DOI:** 10.1371/journal.pone.0009045

**Published:** 2010-02-04

**Authors:** Courtney R. Busch, Jocelyne DiRuggiero

**Affiliations:** Department of Biology and Molecular Genetics, University of Maryland, College Park, Maryland, United States of America; Texas A&M University, United States of America

## Abstract

**Background:**

The genome of the halophilic archaeon *Halobacterium salinarum* NRC-1 encodes for homologs of MutS and MutL, which are key proteins of a DNA mismatch repair pathway conserved in Bacteria and Eukarya. Mismatch repair is essential for retaining the fidelity of genetic information and defects in this pathway result in the deleterious accumulation of mutations and in hereditary diseases in humans.

**Methodology/Principal Findings:**

We calculated the spontaneous genomic mutation rate of *H. salinarum* NRC-1 using fluctuation tests targeting genes of the uracil monophosphate biosynthesis pathway. We found that *H. salinarum* NRC-1 has a low incidence of mutation suggesting the presence of active mechanisms to control spontaneous mutations during replication. The spectrum of mutational changes found in *H. salinarum* NRC-1, and in other archaea, appears to be unique to this domain of life and might be a consequence of their adaption to extreme environmental conditions. In-frame targeted gene deletions of *H. salinarum* NRC-1 mismatch repair genes and phenotypic characterization of the mutants demonstrated that the *mutS* and *mutL* genes are not required for maintenance of the observed mutation rate.

**Conclusions/Significance:**

We established that *H. salinarum* NRC-1 *mutS* and *mutL* genes are redundant to an alternative system that limits spontaneous mutation in this organism. This finding leads to the puzzling question of what mechanism is responsible for maintenance of the low genomic mutation rates observed in the Archaea, which for the most part do not have MutS and MutL homologs.

## Introduction

DNA mismatch repair (MMR) is the major pathway for the repair of DNA replication errors such as nucleotide mismatches, insertions, and deletions [1]. Defects in the MMR pathway lead to genomic instability that can cause a 10 to 1000-fold increase in spontaneous mutability, meiotic defects in eukaryotes, and tolerance to DNA alkylating agents [1], [2], [3]. In humans, inactivation of the MMR pathway leads to a predisposition to hereditary nonpolyposis colon cancer and other types of tumors [2], [3]. The MMR pathway also plays an important role in preventing recombination events between divergent sequences [1], [2].

The key proteins of the MMR pathway, MutS and MutL, are highly conserved between Bacteria and Eukarya. The pathway has been characterized in bacterial and eukaryal systems and comprises three basic steps: (1) MutS/L recognition of mismatch, (2) excision of the mismatched base and surrounding DNA, and (3) repair synthesis [1], [2], [4], [5]. The three-dimensional structure of MutS has been resolved for the *Escherichia coli* and *Thermus aquaticus* proteins [6], [7]. MutS is a 95kDa protein that functions as a dimer *in vivo* [3], [8]. MutS has ATPase activity with Walker A/B sequence motifs and a highly conserved Phe-X-Glu motif responsible for binding DNA [9]. MutL is a 68kDa protein that exists as a dimer in solution and is a member of the Bergerat-fold ATPase/kinase family [3], [10]. Eukaryotes have multiple homologs of the MutS and MutL proteins that form heterodimers suggesting a more complex system than in bacteria with multiple interactions [1], [2]. In contrast to *E. coli* and several other gram-negative bacteria, eukaryotes and most bacteria do not have a methylation-directed MMR system for strand discrimination or a MutH homolog. Studies suggest a nick-directed mechanism using Okazaki fragments produced during replication of the lagging strand or a strand discrimination mechanism directed by the proliferating cell nuclear antigen (PCNA), thus coupling replication and MMR [2], [11].

MMR has not been investigated in the Archaea but studies of the genomic mutation rate in the thermophilic acidophile, *Sulfolobus acidocaldarius*, and the halophile, *Haloferax volcanii*, revealed rates of spontaneous mutation very similar to rates previously reported for DNA-based microorganisms with 3.4×10^−3^ spontaneous mutations per genome, per replication, suggesting that DNA mismatches resulting from DNA replication errors are actively corrected in those organisms [12]. Surprisingly, only 11 out of the 54 archaeal genomes sequenced so far encode for homologs of the conserved MutS1 protein subfamily found in Bacteria and Eukarya [13]. Archaea with MutS1 homologs include halophiles and methanogens, all members of the domain *Euryarchaeota*. These archaeal MutS1 proteins share identical domain structure with their bacterial counterparts and are likely the result of a lateral gene transfer event [13]. Also detected in the Archaea are MutS2-like proteins [13]. In the hyperthermophilic archaeon *Pyrococcus furiosus*, MutS2 has been shown to have ATPase and DNA binding activity but no specific DNA mismatch binding activity [14]. Proteins from the MutS2 subfamily, which are not thought to be involved in MMR, are different in structure and sequence to the MutS1 subfamily proteins except for the MutSAc domain essential for dimerization, ATPase and DNA binding activities [13].

*Halobacterium salinarum* NRC-1 is an extremely halophilic archaeon growing optimally in 4M NaCl [15], [16], [17]. The high osmotic pressure from its environment is counterbalanced by a 4M intracellular concentration of KCl [18]. Previous studies revealed the exceptional resistance of *H. salinarum* NRC-1 to desiccation, UV and ionizing radiation, which was attributed to efficient DNA repair and detoxification systems and to its adaptation to hypersaline environments, characterized by high levels of solar radiation and periodic desiccation [19], [20], [21]. The genome of *H. salinarum* NRC-1 has been sequenced [22] and encodes for proteins of conserved DNA repair pathways that include damage reversal, base excision repair (BER), nucleotide excision repair (NER), homologous recombination, and the bacterial-like MMR proteins MutS and MutL [22]. We demonstrated that the eukaryal-like homologous recombination protein, Mre11, is essential for the repair of DNA double strand breaks in *H. salinarum* NRC-1, whereas Rad50 is dispensable, representing a shift from the eukaryotic model of recombinational repair [23]. Crowley et al. [24] showed that the bacterial NER homologs UvrA/B/C encoded in the genome of *H. salinarum* NRC-1 were essential for the survival of the organism to UV irradiation. These studies demonstrate the mosaic nature of the DNA repair pathways in *H. salinarum* NRC-1, and in the Archaea in general, and raise questions about the nature of DNA MMR in this organism. Through computational analysis we found that *H. salinarum* NRC-1 has three bacterial-like *mutS* genes, a bacterial-like *mutL* gene, 4 bacterial-like *recJ* exonuclease genes, 1 eukaryotic-like *rad2* 5′-3′ exonuclease gene, and a bacterial-like *uvrD* helicase gene, all potentially involved in MMR. Two of the MutS proteins in *H. salinarum* NRC-1, MutS1 and MutS2, are homologous to the MutS1 protein subfamily, and have been renamed MutS1A and MutS1B in this study, while the third MutS protein, MutS3, is homologous to proteins found in the MutS2 subfamily [13]. Whole-genome transcriptomic studies conducted on *H. salinarum* cells exposed to UV and gamma radiation, and to oxidative stress revealed no significant changes in mRNA level for *mutS1A*, *mutS1B*, and *mutL* when compared to untreated cells [20], [21, unpublished].

Here, we used a genetic approach to determine the spontaneous genomic mutation rate in *H. salinarum* NRC-1 and to determine the cellular role of the bacterial-like MMR proteins MutS and MutL encoded in its genome. Our analysis, using fluctuation tests targeting genes of the uridine monophosphate (UMP) biosynthesis pathway, revealed a genomic mutation rate similar to that of other DNA-based microorganisms and a markedly different spectrum of mutational changes. The phenotypic analysis of deletion mutants for the *mutL*, *mutS1A*, *mutS1B*, and *uvrD* genes and a *mutS1A/mutS1B* double mutant showed little difference between the mutant and background strains indicating that the MutS and MutL protein homologs found in *H. salinarum* NRC-1 are not essential for maintaining the low incidence of spontaneous mutations observed in this organism.

## Results

### Genomic Mutation Rate

We calculated the spontaneous genomic mutation rate of *H. salinarum* NRC-1 to determine the replication fidelity in this mesophilic archaeon. We performed six independent fluctuation tests [12], targeting forward mutations in genes of the UMP biosynthetic pathway producing 5-fluoroorotic acid (5-FOA) resistant mutants. The mutation rate was calculated using the equation μ = ln(m/N_av_) [25] and was found to be 3.73×10^−7^+/−1.44×10^−7^ mutations per replication. Sequencing of purified 5-FOA-resistant mutants revealed that only 13 out of 55 sequenced mutants had a mutation in the *pyrF* gene (orotate decarboxylase), only 42 out of 69 had a mutation in the *pyrE2* gene, and none out of 61 had mutation in the *pyrE1* gene (orotate phosphoribosyl transferases). We therefore adjusted the gene mutation rates for the *pyrF* and *pyrE2* genes by factors of 0.24 (13/55) and 0.60 (42/69), respectively, and did not use the *pyrE1* gene in our calculation. The resulting spontaneous mutation rates at the gene level were 8.95×10^−8^ and 2.24×10^−7^ mutations per gene per replication, for the *pyrF* and *pyrE2* genes, respectively. To correct this rate for the fraction of undetected mutations producing no phenotypic effect, we adjusted the total number of base pair substitutions (BPS) using published information on BPS detection efficiency (approximately 0.2) [4]. The resulting rate estimate per gene was calculated as follows:
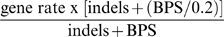
This rate was then converted into a genomic rate by dividing by gene size (*pyrF* = 803bp, and *pyrE2* = 527bp) and multiplying by genome size (2,571,010bp), resulting in an average genomic mutation rate, corrected for undetected mutations, for *H. salinarum* NRC-1 of 1.67×10^−3^±1.4×10^−3^ mutation per replication (0.62×10^−3^ for *pyrF* and 2.7×10^−3^ for *pyrE2*).

### Mutational Spectrum

**Figure 1:**
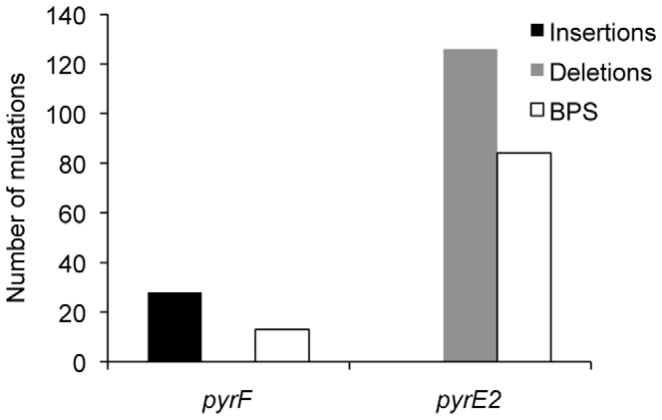
Distribution of mutations in 5-FOA-resistant mutants. Insertions, deletions and base pair substitutions (BPS) in the *pyrF* and *pyrE2* genes were obtained by sequencing 5-FOA-resistant uracil auxotrophs of *H. salinarum* NRC-1.

**Figure 2:**
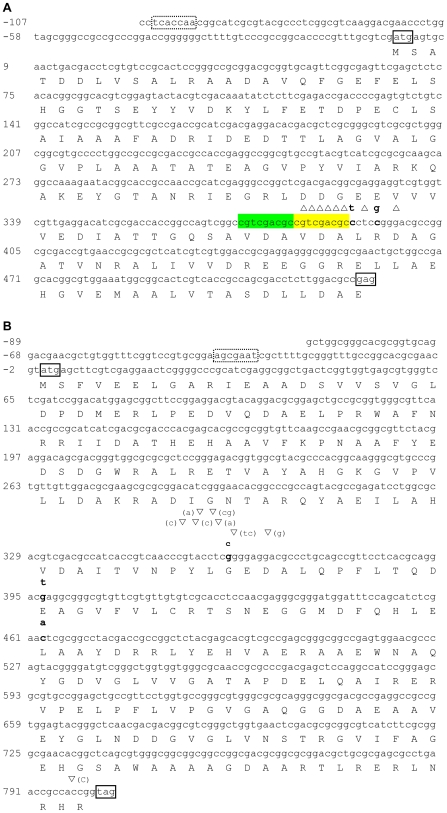
Location of mutations in the *pyrE2* and *pyrF* genes. Mutations were identified by sequencing 5-FOA-resistant mutants. One-letter code for amino acid is under the gene nucleotide sequence; start and stop codons are boxed with solid lines; putative TATA box is boxed with a dotted line; ▿ indicates insertion of the base(s) specified in parenthesis next to the symbol; ▵ indicates deletion of bases directly located below the symbol; BPS changes are indicated above the sequence in bold; highlighted in green and yellow in *pyrE2* are the two 9-nt direct repeats. (A) *pyrE2* gene and (B) *pyrF* gene.

One hundred forty-nine 5-FOA-resistant mutants were recovered from two fluctuation tests and of those 55 were sequenced using primers for *pyrF*, 61 using primers for *pyrE1*, and 65 using primers for *pyrE2*. Mutations were only found in the coding regions of the *pyrF* and *pyrE2* genes and a number of mutants had mutations in more than one gene (Table 1). No mutation was found within a 100-bp region upstream of either gene. A disproportionate number of deletions were found in the *pyrE2* gene when compared to the *pyrF* gene (Figure 1, Table 2). All of the *pyrE2* mutations occurred at a single hotspot in the gene at positions 382–397, with a 7-nucleotide (nt) deletion (GTCGACG) found in 42 mutants; two 1-nt deletions and two single BPS also contributed to the changes observed in this gene (Table 2, Figure 2A). A 9-nt sequence found at the *pyrE2* gene mutational hotspot was the direct repeat of a sequence located immediately upstream (Figure 2A). Mutations were distributed throughout the *pyrF* gene with a concentration of insertions at position 354–365 (Table 2, Figure 2B). None of the mutations were the result of a transposon element insertion. Indels out numbered BPS and approximately 80% of the BPS resulted in non-synonymous amino acid changes. BPS found in the *pyrF* and *pyrE2* genes were mostly transversions (80%) (Table 2).

**Table 1:** 5-FOA-resistant uracil auxotrophs of *H. salinarum* NRC-1 with mutations in multiple UMP biosynthetic genes.

		Mutations(1)		
Genes analyzed	# of clones sequenced	*pyrF*	*pyrE2*	none
*pyrF*	4	2	1	1
*pyrE1*				
*pyrE2*				
*pyrF*	19	5	nd	14
*pyrE1*				
*pyrF*	16	1(2)	15	1
*pyrE2*				
*pyrE1*	8	nd	4	4
*pyrE2*				

(1) no mutation in *pyrE1*.

(2) mutation in both *pyrF* and *pyrE2*.

nd not determined.

**Table 2:** Types and positions of spontaneous mutations in the *pyrF* and *pyrE2* genes.

Gene position (bp)	Number of independent isolates with this mutation	Type of mutation(1)
***pyrF***		
354	4	Insertion (C)
356	4	Insertion (C)
357	4	Insertion (A)
359	4	Insertion (CG)
360	1	Insertion (A)
361	4	G→C(2)
362	4	Insertion (TC)
365	4	Insertion (G)
397	8	G→T(2)
463	1	C→A(2)
798	3	Insertion (C)
***pyrE2***		
382	42	Deletion (GTCGACG)
390	42	C→T
392	42	Deletion (T)
394	42	C→G(2)
397	42	Deletion (G)

(1) insertion indicated was found prior to the stated base pair position.

(2) non-synonymous base pair changes.

### MutS and MutL Are Not Essential for the Low Incidence of Mutation Observed in *H. salinarum* NRC-1

**Figure 3:**
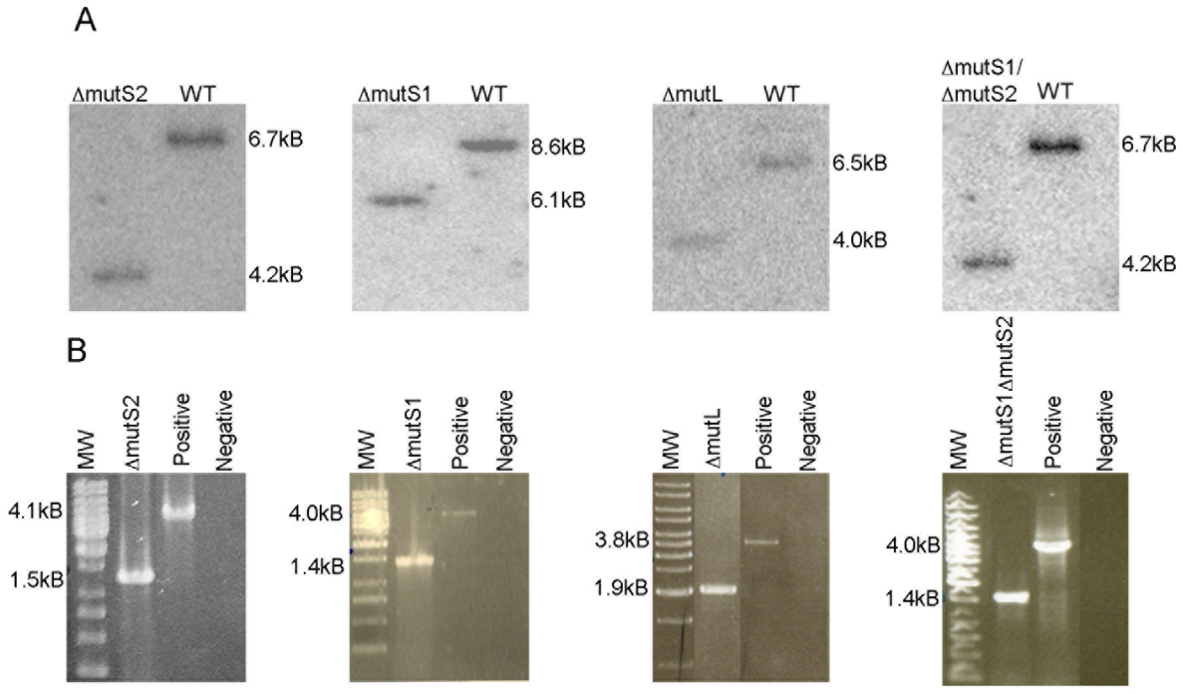
Analysis of deletions in the *mutL*, *mutS1(mutS1A)*, *mutS2 (mutS1B)*, double *mutS*, and *uvrD* genes. Probes for Southern blot analysis were designed to hybridize to regions 500 nt downstream of the target genes coding region. PCR analysis primers were located 500 nt upstream of the start codon of the targeted gene and 1000 nt downstream of the stop codon. (A) Southern hybridizations. (B) PCR analysis: positive lanes template was wildtype *H. salinarum* NRC-1 DNA, negative lanes had no template DNA.

**Figure 4:**
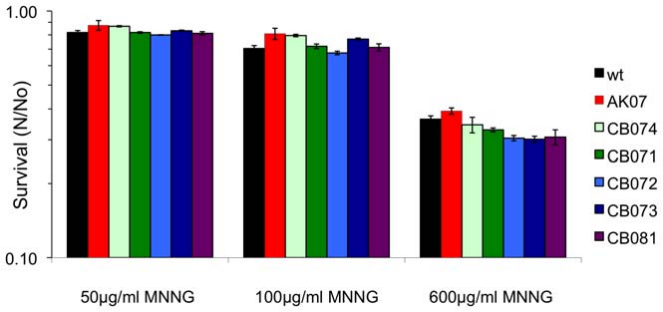
Survival of *H. salinarum* NRC-1 background and mutant strains to MNNG. *H. salinarum* NRC-1 background strain Δ*ura3* (AK07) and mutant strains Δ*mutL* (CB074), Δ*mutS1A* (CB071), Δ*mutS1B* (CB072), Δ*mutS1A*Δ*mutS1B* (CB073), and *ΔuvrD* (CB081) were exposed to 50, 100, and 600mg/L of MNNG. Survival was calculated as the average ratio (N/No) of surviving CFU from treated cultures (N) and untreated (No) cultures. Data are the average of a least three independent experiments, with standard errors shown.

To determine whether the bacterial-like MMR proteins encoded in the genome of *H. salinarum* NRC-1 were essential in maintaining the low genomic mutation rate we observed, we carried out targeted gene deletions of the *mutS1A*, *mutS1B*, *mutL*, and *uvrD* genes, with a double deletion of the *mutS1A* and *mutS1B* genes, using the background strains AK07 (Δ*ura3*) and CB08 (Δ*ura3*Δ*zim*) [26], [27]. Genotypes of mutant strains CB071 (Δ*mutS1A*), CB072 (Δ*mutS1B*), CB073 (Δ*mutS1AΔmutS1B*), CB074 (Δ*mutL*), and CB081 (Δ*uvrD*) were confirmed by Southern blot hybridization after initial screening by PCR (Figure 3). Phenotypic characterization of the mutant strains revealed no growth defects at 37, 42, and 45°C when compared to the background strain AK07 (Δ*ura3*). While tolerance to alkylating agents is a hallmark of bacterial and eukaryal MMR systems, we found no increased tolerance to alkylation when the mutants were exposed to 600mg/L N-Methyl-N′-Nitro-N-Nitrosoguanidine (MNNG) (Figure 4). Mismatch repair deletion mutants were also constructed in another background strain, CB08 (Δ*ura3*Δ*zim*), to confirm the validity of our gene deletions. All mismatch deletion mutants in the Δ*ura3*Δ*zim* background showed the same phenotypes as the mutants constructed in the Δ*ura3* background, which together with PCR and Southern blot analyses established that we constructed true gene deletions.

Significant increases in mutation rate following deletion of mismatch repair genes has been demonstrated in both Bacteria and Eukarya [1], [2], [3]. Here we designed a 5-FOA mutation frequency assay to test whether our *H. salinarum* NRC-1 MMR deficient strains showed increased mutation rates. This assay, targeting forward mutations in a plasmid copy of the *pyrF* (*ura3*) gene, allowed us to compare the mutation frequencies for each of the mutant strains tested with that of the background strain. The use of a plasmid assay was born out of necessity because our MMR deletions were constructed in a *pyrF* deletion background; the limited genetic markers available for *H. salinarum* NRC-1 constrained our ability to restore the *pyrF* gene into the genome of *H. salinarum* NRC-1. Using fluctuation tests, we obtained comparable mutation frequencies (ratio of 5-FOA-resistant colonies to the average number of colonies plated) for the background and deletion mutant strains (Table 3). Sequencing of 20 mutants for strain AK07 (Δ*ura3*), 22 mutants for strain CB071 (Δ*mutS1A*), 19 mutants for strain CB073 (Δ*mutS1A*Δ*mutS1B*), and 33 mutants for strain CB074 (Δ*mutL*) showed that approximately 70% of the mutants had changes in the plasmid copy of the *pyrF* gene and that the distribution of BPS, insertions and deletions in the *pyrF* gene of the MMR mutants was similar to that found for the control strain AK07 (Table 3). We found that all the changes in the plasmid copies of the *pyrF* gene were BPS, with the exception of the insertion of a T in one of the CB071 (Δ*mutS1A*) mutants, and that most BPS were C/G or G/C transversions (55 to 75%).

**Table 3:** Mutation frequencies and mutation types found in plasmid copies of the *pyrF* gene in MMR mutant and background strains.

Strains names	Strains phenotypes	Mutation frequencies ×10^5^	# of deletions	# of insertions	# of BPS
AK07	Δ*ura3*	5.70±4.55	0	0	22
CB071	Δ*ura3*Δ*mutS1A*	8.99±5.72	1	0	22
CB073	Δ*ura3*Δ*mutS1A*Δ*mutS1B*	8.00±5.39	0	0	18
CB074	Δ*ura3*Δ*mutL*	7.39±4.39	0	0	17

## Discussion

The MMR pathway is essential for maintaining genome stability by correcting errors introduced by DNA polymerases during DNA replication [1], [2], [3]. We determined the genomic mutation rate of the halophilic mesophile, *H. salinarum* NRC-1, one of the few archaea to encode for a bacterial-like version of the conserved MutS and MutL proteins. We found that *H. salinarum* NRC-1 mutation rate was similar to that previously calculated for DNA-based microorganisms, with 1.67×10^−3^ mutations per genome per replication, suggesting the presence of high fidelity replication machinery in this organism [4], [12], [28], [29], [30], [31], [32]. The DNA-based organisms that formed the basis for this genomic mutation rate comparison are all mesophiles, belong to the Bacteria and the Eukarya domains, and include several bacteriophages. When we compared the mutation rate of *H. salinarum* NRC-1 with that of organisms adapted to high temperature, we found that it was almost twice that of the acidophilic archaeon, *S. acidocaldarius*, and more than an order of magnitude higher than that of the bacterium *Thermus thermophilus*, with 1.8×10^−3^ and 9.7×10^−4^ mutations per genome per replication, respectively [4], [33]. While the low genomic mutation rate found in thermophiles might be an adaption to extreme temperature conditions [33], the mutation rate of the mesophilic halophile, *H. volcanii* was also found to be extremely low with 4.5×10^−4^ mutations per genome per replication [5]. It is possible that this extremely low genomic mutation rate is the result of phenotypic lag or the effects of *H. volcanii* polyploidy [5]. However, *H. salinarum* NRC-1, similarly to *H. volcanii*, has approximately 20 copies of its chromosome per cell [5], [34] suggesting that high gene redundancy does not necessarily result in mutations not being efficiently detected if they occur at low copy number.

*H. volcanii* has an unusual mutational spectrum with a prevalence of in-frame indels flanked by direct repeats [5]. In *H. salinarum* NRC-1, a 9-nt direct repeat in the *pyrE2* gene resulted in a mutational hotspot that strongly biased the mutational spectrum of this gene toward deletions. Short direct repeats, tandem repeats and monotonic runs are known to promote strand misalignments during DNA replication and might explain the increased deletion frequency we observed in the *H. salinarum* NRC-1 *pyrE2* gene [35]. An alternative explanation might be that this 7-nt deletion and the 4 other mutations found in the *pyrE2* gene – shared by 42 of the mutants - were a pre-existing set of mutations present in the initial cultures used for the fluctuation tests, which were a subset of the 6 fluctuation tests performed to calculate the genomic mutation rate of *H. salinarum* NRC-1. With the exception of the *pyrE2* gene hotspot most of the indels found in *H. salinarum* NRC-1 were ±1 frameshifts, which were also found to be predominant in mesophiles and in the two thermophilic organisms investigated so far [4], [33]. A low overall number of BPS was found in *H. salinarum* NRC-1, in the two archaea, *S. acidocaldarius* and *H. volcanii*, and in the thermophilic bacterium, *T. thermophilus* [4], [5], [33]. This is a departure from data reported for mesophilic bacteria and eukaryotes where BPS constituted the overwhelming majority, 60–80%, of mutations [4], [12]. The low occurrence of BPS in Archaea might be related to the high GC content (>60%) found in both *H. salinarum* NRC-1 and *H. volcanii*, which results in long runs of G and C nucleotides [22]. During replication these GC runs may cause polymerase slippage leading to the formation of indels. However, *S. acidocaldarius* only has a 36% GC content arguing against this idea [36]. An alternative explanation for the low BPS found in these archaea might be a consequence of their adaptations to extreme environmental conditions – high temperature and high salt – resulting in intrinsic properties of their replication machinery [37], [38], [39]. This argument is strengthened by the recent finding of a low mutation rate and a low incidence of BPS in the thermophilic bacterium, *T. thermophilus* [33].

Phenotypic analyses of MMR deletion mutants in *H. salinarum* NRC-1 did not show an increase in alkylation tolerance to MNNG. This was in contrast to previous studies showing that increased alkylation tolerance is a hallmark of MMR systems in most bacteria and eukarya [2], [40]. However, in *Saccharomyces cerevisiae* no tolerance to MNNG was demonstrated in MMR deficient yeast strains unless the *MGT1* methyltransferase, responsible for correcting O^6^-methyl guanine damage, was also absent [41]. The *H. salinarum* NRC-1 genome does not encode for a yeast MGT1 methyltransferase homolog but this does not rule out the repair of O^6^-methyl guanine lesions by another glycosylase yet to be identified.

An increased mutation rate following deletion of mismatch repair genes has been demonstrated both in Bacteria and Eukarya. In *D. radiodurans* and *E. coli*, the mutation rate was calculated in cells deficient in MutS, MutL, or UvrD proteins and was found to increase 7 to 1,000-fold compared to wildtype cells [42], [43]. In *E. coli* lacking MutH, MutS, and MutL proteins, forward mutation studies in the *lacI* gene showed a 200-fold increase in mutation rate [32], [44], [45]. In *S. cerevisiae*, both forward and reverse mutation rate studies have shown that the MutS and MutL homologs are required for base correction [1], [2]. In contrast to those studies, the mutation frequencies obtained from our MMR deletion mutants showed that the bacterial-like MMR pathway encoded in the genome of *H. salinarum* NRC-1 is not essential for maintaining the low genomic mutation rate we observed in this organism. We found that the mutational spectrum of *H. salinarum* NRC-1 MMR mutants is identical to that of the control strain, indicating that if functional, the bacterial-like MMR system does not have a major role in this organism. This finding is a departure from the reported role of the bacterial-like NER pathway in *H. salinarum* NRC-1, where the *uvrA*, *uvrB* or *uvrC* genes were required for the repair of photoproducts induced by UV light [24].

No homologs of the conserved mismatch repair genes, *mutS* and *mutL*, have been found in the genome of *S. acidocaldarius* despite its low genomic mutation rate, leading to the puzzling question of what is responsible for the maintenance of the low genomic mutation rate observed in this organism and in the Archaea as a whole [4], [5], [46]. One explanation might come from the adaptive mechanisms these organisms have evolved to thrive in extreme environments. Studies calculating replication fidelity showed that the polymerase from *P. furiosus* has 10-fold higher replication fidelity than the polymerase from *T. aquaticus*, a thermophilic bacterium, and 5-fold higher replication fidelity than *E. coli* DNA polymerase III holoenzyme [37], [38], [39], [47], [48]. The replicative polymerases in the Archaea are members of the B-family and are more similar to eukaryotic polymerases than to bacterial ones [49]. The higher fidelity of archaeal polymerases, resulting from differences in sequence and structure, could play a role in maintaining the genomic integrity of the Archaea. An alternative hypothesis might be that MMR is carried out by DNA repair enzymes specifically recruited to this function rather than by a canonical MMR pathway [50]. For example, deamination of cytosine to uracil in a GC base pair is a major mutagenic event that generates a G.C to A.T mutations [51]. Polymerases in the Archaea possess the unique ability to stall when a uracil residue is encountered [52], the uracil is then removed by an uracil-DNA glycosylase [52], [53]. Furthermore, direct interaction between uracil-DNA glycosylase and a PCNA homolog from *Pyrobaculum aerophilum* has been documented [54], [55], [56], suggesting the possibility of recruitment of DNA glycosylases, or other DNA repair proteins, to damage sites by the replication machinery.

Further studies, in particular to elucidate if there is any MMR activity in the protein complement of *H. salinarum* NRC-1, are necessary to differentiate if the low mutation rate observed in this organism is the result of intrinsic properties of its DNA polymerase or if DNA repair activities are recruited to the replication site to remove mismatches. The presence of bacterial-like MMR genes in several archaea is thought to be the result of lateral gene transfer from bacteria [13], [20] and, although we showed here that the *mutS* and *mutL* genes are not essential for the maintenance of a low mutation rate in *H. salinarum* NRC-1, it does not mean that this pathway is not functional, existing possibly as an alternate or minor pathway. Until the mechanism(s) maintaining genomic stability in the archaea are elucidated, this hypothesis remains difficult to test.

## Materials and Methods

### Organism and Growth Conditions

*Halobacterium salinarum* NRC-1 (ATCC 700922) was grown in standard GN101 medium (250g/L NaCl, 20g/L MgSO_4_, 2g/L KCl, 3g/L sodium citrate, 10g/L Oxoid brand bacteriological peptone), pH 7.2, with the addition of 1 mL/L trace elements solution (31.5mg/L FeSO_4_·7H_2_O, 4.4mg/L ZnSO_4_·7H_2_O, 3.3mg/L MnSO_4_·H_2_O, 0.1mg/L CuSO_4_·5H_2_O) at 42°C shaking in a Gyromax 737 shaker (Amerex Instruments; LaFayette, CA) at 220rpm. When specified, the GN101 media was supplemented with 50mg/L uracil and 350mg/L 5-fluoroorotic acid (5-FOA) (Sigma; St. Louis, MO), final concentrations. Basal salts solution (BSS), of the same composition as GN101 without the peptone, was used for culture dilutions.

### Targeted Gene Deletion

In-frame gene deletions, Δ*mutL* (CB074), Δ*mutS1A* (CB071), Δ*mutS1B* (CB072), Δ*mutS1A*Δ*mutS1B* (CB073), and Δ*uvrD* (CB081), were constructed as described previously [26], [27]. In short, uracil dropout medium was used for selection for uracil prototrophy following transformation of strain AK07 (Δ*ura3*) [23] and strain CB08 (Δ*ura3*Δ*zim*) (DiRuggiero *et al.* unpublished) with plasmid pNBK07 (from M.P. Krebs, Illinois State University, Normal, Illinois) bearing the knockout gene constructs and the *ura3* marker for uracil biosynthesis. The *zim* gene was originally thought to have a function similar to the *dam* methylase from *E. coli*. Further experiments showed that it is a putative CTAG methylase from an uncharacterized restriction and modification system. 5-FOA was subsequently used to select intra-molecular recombinants that lost the plasmid. Recombinants were screened by PCR and confirmed by Southern blot analysis. The GN101 medium was supplemented with 50mg/L uracil for all Δ*ura3* strains.

### Mutation Rate Assay and Mutant Isolation

Fluctuation tests were performed as previously described [4], [57] with modifications. Tubes of GN101 medium supplemented with 50mg/L uracil and inoculated with one colony of *H. salinarum* NRC-1 each were incubated at 42°C, with shaking, for two days. The resulting cultures were diluted to 1×10^2^ cells/mL, 150µL aliquots were dispensed into 60 wells of a 96 well flat-bottomed plate (VWR; West Chester, PA), and incubated for 3 days at 42°C without shaking until cell density reached approximately 1×10^5^ cells/mL. The entire contents from each well were spread on GN101 plates with 50mg/L uracil and 350mg/L 5-FOA and colonies were counted 11 days after incubation at 42°C. The spontaneous mutation rate for *H. salinarum* NRC-1 was calculated using the relationship μ = ln(m/N_av_) [4], [5]. The mutation rate, μ, is equal to the natural log of m, number of mutational events per culture, divided by N_av_, average number of cells per culture. The m value was calculated using the MSS Maximum-Likelihood method as previously described [25]. Colonies from the 5-FOA plates were randomly selected, clonally purified by restreaking twice onto GN101 plates with 50mg/L uracil and 350mg/L 5-FOA and stored at −80°C for sequence analysis.

### Sequence Analysis

The UMP biosynthesis genes, *pyrE1*, *pyrE2*, and *pyrF*, were amplified by colony PCR using clonally purified 5-FOA resistant mutants as described above and the following primers: pyrE1-F (5′CCTCGTCCTGGAGAACAAAG3′), pyrE1-R (5′ATCGAAGGCCATGTCCCACCGT3′), pyrE2-F (5′GGTTCATACCGACCACACG3′), pyrE2-R (5′TCGGCGACACCTTCGGGCTG3′), pyrF-F (5′GCGCGCCTCGTGGTGTTCGT3′), and pyrF-R (5′AGCGTCGTCTGTGACACCCA3′). Primers were located 200 bp upstream and 100 downstream of the coding region for these genes. PCR conditions were 2 minutes at 94°C, 30 cycles of 35 seconds at 94°C, 40 seconds at 56°C, and 60 seconds at 72°C, and a final 5 minutes at 72°C using FastTaq DNA polymerase (Roche; Indianapolis, IN). PCR products were purified using ExoSap-IT (GE Healthcare; Piscataway, NJ) and sequencing was performed using an Applied Biosystems 3730xl DNA Analyzer with BigDye Terminator reagents (Applied Biosystems; Foster City, CA).

### Mutation Frequency Assay by the 5-FOA Selection Method

The *ura3* (*pyrF*) gene and its native promoter were PCR amplified from *H. salinarum* NRC-1 genomic DNA, cloned into plasmid pNBPA, and the resulting construct was transformed into strain AK07 (Δ*ura3*) and into the deletion mutant strains CB071 (Δ*mutS1A*), CB073 (Δ*mutS1A*Δ*mutS1B*), and CB074 (Δ*mutL*). Fluctuation tests were performed as described above and cultures were plated *in toto* on GN101 medium supplemented with 50mg/L uracil, 50µM mevinolin, and 350mg/L 5-FOA. Colonies were screened by PCR to ensure presence of the *ura3* gene on plasmid pNBPA and the *ura3* gene was sequenced using primers: ura3-Acc65I (5′GCGGGTACCGTCGGCTGGCGGGCACGCGGT3′) and ura3-SpeI (5′GCGACTAGTCTACCGGTGGCGGTTCAGGCG3′). Mutation frequency was calculated as the ratio of 5-FOA-resistant colonies to the average number of colonies plated.

### Survival Assays

#### Temperature growth defect assay

Single colonies of wildtype *H. salinarum* NRC-1 and deletion mutant strains were grown to mid-log phase in GN101 medium supplemented with 50mg/L uracil. Cultures were diluted to 10^4^, 10^3^, and 10^2^ cells/mL and 5µL of each spotted in triplicate on GN101 medium supplemented with 50mg/L uracil. Plates were observed for growth at 37°C, 42°C, and 45°C after 7 days of incubation.

#### N-Methyl-N′-Nitro-N-Nitrosoguanidine (MNNG) survival assay

cultures were grown to OD_600_ 0.6 in GN101 medium supplemented with 50mg/L uracil and diluted to OD_600_ 0.4. Cultures were divided into 5mL aliquots, treated with the addition of 0 or 600mg/L MNNG, in triplicate, and incubated in the dark at 42°C with shaking for 1 hour. Cells were washed, diluted in BSS, and plated on GN101 medium supplemented with 50mg/mL uracil. Plates were incubated at 42°C in the dark for 7–10 days and colonies counted. Survival was calculated as N/No where N is the number of viable cells after MNNG treatment and No is the number of viable cells without treatment. MNNG (TimTech; New Zealand) stock solution was made at 50g/L in DMSO.
